# Implication of affective traits in bipolar vulnerability: A study of university students

**DOI:** 10.1192/j.eurpsy.2026.11195

**Published:** 2026-07-31

**Authors:** N. Regaieg, H. Mezghani, F. Guermazi, R. Masmoudi, I. Feki, J. Masmoudi, I. Baati

**Affiliations:** Psychiatry A Department, Hedi Chaker University Hospital, Sfax, Tunisia

## Abstract

**Introduction:**

Emotional fluctuations and emotional hyperreactivity characterize bipolar disorder. These affective patterns are often linked to enduring personality traits. Gaining a better understanding of the relationship between affective temperaments and bipolar vulnerability facilitates the identification of those at risk.

**Objectives:**

The objective of this study is to investigate the relationship between affective temperaments and bipolar vulnerability among students at the Institute of Arts and Crafts in Sfax, Tunisia.

**Methods:**

Our study was cross-sectional, descriptive, and analytical, conducted from February 1^st^ to March 5^th^, 2025, among students at the Institute of Arts and Crafts in Sfax (Tunisia). These students were assessed using an anonymous self-administered questionnaire including sociodemographic, clinical, and additional data, as well as two psychometric scales in their Arabic versions: the Tunisian TEMPERAMENT Scale (TEMPS) to measure affective temperaments and the Mood Disorder Questionnaire (MDQ), used for screening for bipolar disorder.

**Results:**

The study included 60 students, with a sex ratio of 0.6. The median age of the sample was 20 years [19-22 years]. According to the MDQ scale, 28.3% of the sample had a positive score, indicating a possible screening for bipolar disorder.

Among these students with a positive MDQ score, 66.3% reported difficulties controlling their emotions, with frequent changes between periods of high energy and moments of emotional weakness, and 41.7% of students indicated having made impulsive decisions during periods of intense emotions, often marked by excess energy or a state of excitement.

Moreover, 56.7% of these students reported experiencing periods where they felt unusually euphoric, hyperactive, or particularly sad for no apparent reason.

Our study showed that students with a positive MDQ score had significantly higher cyclothymic and hyperthymic temperament scores than those with a negative MDQ score (p < 0.001).

Conversely, no significant association was found between a positive screening result for bipolar disorder and scores attributed to depressive, anxious or irritable affectives temperaments.

**Conclusions:**

Understanding how affective temperaments interact with vulnerability to mood disorders in artistic students could improve prevention and early management of the disorder.

**Disclosure of Interest:**

None Declared

